# Pyrazinamide Susceptibility and *pncA* Mutation Profiles of Mycobacterium tuberculosis among Multidrug-Resistant Tuberculosis Patients in Bangladesh

**DOI:** 10.1128/AAC.00511-17

**Published:** 2017-08-24

**Authors:** Arfatur Rahman, Sara Sabrina Ferdous, Shahriar Ahmed, S. M. Mazidur Rahman, Mohammad Khaja Mafij Uddin, Suporn Pholwat, Jean Gratz, Eric Houpt, Sayera Banu

**Affiliations:** a Mycobacteriology Laboratory, Infectious Diseases Division, International Center for Diarrheal Disease Research, Dhaka, Bangladesh; b Division of Infectious Diseases and International Health, Department of Medicine, University of Virginia, Charlottesville, Virginia, USA

**Keywords:** pyrazinamide, Mycobacterium tuberculosis, MDR-TB, *pncA*, Bangladesh

## Abstract

Pyrazinamide (PZA) is a frontline antituberculosis (anti-TB) drug used in both first- and second-line treatment regimens. However, due to complex laboratory requirements, the PZA susceptibility test is rarely performed, leading to a scarcity of data on susceptibility to PZA. Bangladesh is a country with a burden of high rates of both TB and multidrug-resistant TB (MDR-TB), but to our knowledge, published data on rates of PZA susceptibility (PZA^s^), especially among MDR-TB patients, are limited. We aimed to analyze the PZA susceptibility patterns of Mycobacterium tuberculosis isolates from MDR-TB patients and to correlate the *pncA* mutation with PZA resistance in Bangladesh. A total of 169 confirmed MDR M. tuberculosis isolates from a pool of specimens collected in a nationwide surveillance study were included in this analysis. All the isolates were tested for phenotypic PZA susceptibility in Bactec mycobacterial growth indicator tube (MGIT) culture medium, and the *pncA* gene was sequenced. We also correlated different types of clinical information and treatment outcomes with PZA susceptibility. We found that 45% of isolates were phenotypically PZA resistant. Sequencing of the *pncA* gene revealed a high concordance (82.2%) between the *pncA* gene sequence and the phenotypic assay results. A total of 64 different mutations were found, and 9 isolates harbored multiple mutations. We detected 27 new *pncA* mutations. We did not find any significant correlation between the different clinical categories, the genetic lineage, or treatment outcome group and PZA susceptibility. Considering the turnaround time, sequencing would be the more feasible option to determine PZA susceptibility, and further studies to investigate the MIC of PZA should be conducted to determine an effective dose of the drug.

## INTRODUCTION

Pyrazinamide (PZA) is a frontline antituberculosis (anti-TB) drug used in both first- and second-line treatment regimens (1, 2). It is an unconventional and paradoxical drug that shows no activity against growing bacilli but has the unique ability to combat persister bacilli that are sequestered within macrophages and not killed by other drugs (3–5). Because of its unique sterilizing activity, the use of PZA in combination with isoniazid (INH) and rifampin (RIF) shortens the treatment regimen from 9 to 12 months to 6 months (3, 6, 7).

Pyrazinamide is a prodrug that is converted into its active form, pyrazinoic acid (POA), by the enzyme pyrazinamidase (PZase), encoded by the *pncA* gene (6). Intracellular accumulation of POA causes acidification of the cytoplasm, depletion of the membrane potential, and inhibition of various targets, which ultimately lead to bacterial death (8–10). There are many factors behind PZA resistance (PZA^r^), but on average, about 72 to 98% of cases are due to mutations in the *pncA* gene (6, 8, 11–13). More than hundreds of mutations occur in the *pncA* gene and its upstream regulatory region (14). The mutation types include the substitution of nucleotides and the insertion and deletion of single or multiple nucleotides in the coding and upstream promoter regions of the *pncA* gene. These mutations are highly diverse and scattered along the gene. Besides mutations in the *pncA* gene, there are alternative mechanisms of PZA^r^, as demonstrated by the presence of a wild-type (WT) *pncA* gene in PZA^r^ Mycobacterium tuberculosis (12, 15, 16). Few studies have shown that mutations in the *panD* and *rpsA* genes are also linked to PZA^r^ (12, 17, 18).

Though the prevalence of PZA^r^ is higher in multidrug-resistant (MDR) TB (MDR-TB) cases, due to technical difficulties, the phenotypic PZA susceptibility test is rarely performed (19). PZA is active only at low pH, and thus, it requires acidic culture medium for susceptibility testing. The acidic nature of the culture medium prevents about 20 to 25% of isolates from growing (19). On the other hand, a large inoculum size (over 10^7^ cells/ml) can cause alkalization of the medium, leading to false PZA^r^ (20). Due to the technical challenges in the laboratory and the possibility of false susceptibility, most TB laboratories do not perform PZA susceptibility testing (19, 21). As a result, most patients infected with PZA^r^ strains fail to get appropriate treatment (22). This is also the main reason behind the scarcity of data regarding PZA susceptibility. Though PZA is used in MDR-TB treatment, very few data regarding the prevalence of PZA^r^ among MDR-TB patients in Bangladesh are available. Few collaborative studies have used M. tuberculosis isolates from Bangladesh and shown the PZA susceptibility status of those isolates. A recent multicenter study has shown a rate of PZA^r^ of about 5.1% among isolates from patients with pulmonary TB and 36.7% among isolates from patients with RIF-resistant TB from Bangladesh (23). Also, another study used MDR M. tuberculosis isolates from Bangladesh and compared the results of the pyrazinamidase assay with the *pncA* gene mutations found (11). Besides, different studies from around the world have shown that roughly 2 to 7.5% of non-MDR-TB cases, 36 to 85% of MDR-TB cases, and about 16% of all TB cases are caused by PZA^r^ isolates (14, 24–26).

Bangladesh is a country with a burden of high rates of both TB and MDR-TB. Very recently, the shorter regimen has been recommended as the standard of care for MDR-TB throughout Bangladesh. Although in most MDR-TB treatment centers, the World Health Organization (WHO) standard 24-month regimen is still used for the treatment of MDR-TB, the whole country will migrate to the shorter regimen within a year or 2. Both of the MDR-TB regimens include PZA along with other drugs. As there is no approved test for the determination of PZA susceptibility, testing is not an essential prerequisite for starting treatment (27). However, if PZA^r^ could be determined by reliable susceptibility testing before treatment initiation, clinicians would be able to reconsider the treatment regimen (27). In the *National Guidelines and Operational Manual for Programmatic Management of Drug Resistant TB* (28), it is also mentioned that PZA should be used with caution and should not be relied upon as a key drug. For diagnosis and susceptibility testing of isolates from patients with TB and MDR-TB, various conventional and molecular methods are being used in Bangladesh (29, 30). The Xpert MTB/RIF (Cepheid, Sunnyvale, CA) assay is widely used for the rapid detection of MDR-TB. However, the use of GenoType MTBDR*plus* (Hain Life Science, Nehren, Germany) is limited to the National Tuberculosis Reference Laboratory and Mycobacteriology Laboratory of the International Center for Diarrheal Disease Research, Bangladesh (ICDDR,B). To our knowledge, the capacity to perform PZA susceptibility testing is lacking in Bangladesh. As a result of this and also as the national guideline does not require it, susceptibility testing before treatment initiation is not performed in practice. The aim of this study was to address the pattern of susceptibility to PZA among isolates from MDR-TB patients in Bangladesh and compare that pattern with the results of phenotypic and genotypic assays as well as the *pncA* mutation profile. Also, we aimed to demonstrate a correlation between the PZA susceptibility testing result and the clinical outcome of the treatment.

## RESULTS

### PZA susceptibility pattern and patient and M. tuberculosis characteristics.

All the isolates (*n* = 169) included in this analysis were confirmed to be MDR by testing for susceptibility to four first-line anti-TB drugs on Lowenstein-Jensen (LJ) medium. The phenotypic PZA assay in liquid medium revealed that 76 isolates (45%; 95% confidence interval [CI], 37.7 to 52.5%) were PZA^r^. Among these MDR M. tuberculosis isolates, 122 were resistant to all four drugs and 64 (52.5%; 95% CI, 43.7 to 61.1%) of them were PZA^r^. The frequency of PZA^r^ was higher among MDR isolates with ethambutol (EMB) resistance (61.5%; 95% CI, 35.5 to 82.3%) than among isolates with sole INH and RIF resistance (6.7%; 95% CI, 1.2 to 29.8%) and MDR isolates with sole streptomycin (STR) resistance (15.8%; 95% CI, 5.5 to 37.6%) (Table 1). Because of the possibility of a false PZA susceptibility result in a mycobacterial growth indicator tube (MGIT) due to an inoculum effect, we repeated the phenotypic PZA susceptibility test for 16 isolates (approximately 10%, 10 PZA^r^ isolates and 6 PZA^s^ isolates). Except for one isolate, the results of repeat testing were consistent with those of the previous test. Only one PZA^r^ isolate was found to be PZA^s^ in the repeat test.

**TABLE 1:** PZA susceptibility pattern among different participant and M. tuberculosis categories

Characteristic	PZA^s^		PZA^r^	
	No. (%) of participants	95% CI	No. (%) of participants	95% CI
Age (yr)				
0–20	16 (44.4)	29.5–60.4	20 (55.6)	39.6–70.5
21–40	53 (55.2)	45.3–64.8	43 (44.8)	35.2–54.7
41–60	20 (60.6)	43.7–75.3	13 (39.4)	24.7–56.3
>60	4	100	0	0
Sex				
Male	58 (58.6)	48.7–67.8	41 (41.4)	32.2–51.3
Female	35 (50.0)	38.6–61.4	35 (50.0)	38.6–61.4
History of TB				
Yes	88 (56.4)	48.6–63.9	68 (43.6)	36.1–51.4
No	5 (38.5)	17.7–64.5	8 (61.5)	35.5–82.3
TB exposure history				
Yes	11 (52.4)	32.4–71.7	10 (47.6)	28.3–67.6
No	82 (55.4)	47.4–63.2	66 (44.6)	36.8–52.6
TB patients in the family				
Yes	18 (47.4)	32.5–62.7	20 (52.6)	37.3–67.5
No	75 (57.3)	48.7–65.4	56 (42.7)	34.6–51.3
Previous treatment outcome (*n* = 156)				
Cured/treatment completed	29 (56.9)	43.3–69.5	22 (43.1)	30.5–56.7
Defaulted	9 (64.3)	38.8–83.6	5 (35.7)	16.4–61.2
Failed new patient regimen using first-line drugs only	46 (60.5)	49.3–70.7	30 (39.5)	29.3–50.7
Failed regimen including second-line drugs	3 (21.4)	7.6– 47.6	11 (78.6)	52.4–92.4
Unknown	1	100	0	0
Drug resistance pattern				
STR, INH, RIF, and EMB	58 (47.5)	38.9–56.3	64 (52.5)	43.7–61.1
INH and RIF	14 (93.3)	70.2–98.8	1 (6.7)	1.2–29.8
INH, RIF, and STR	16 (84.2)	62.4–94.5	3 (15.8)	5.5–37.6
INH, RIF, and EMB	5 (38.5)	17.7–64.5	8 (61.5)	35.5–82.3
M. tuberculosis type				
Beijing	30 (53.6)	40.7–66.0	26 (46.4)	34–59.3
Non-Beijing	63 (55.8)	46.6–64.6	50 (44.2)	35.4–53.4
TbD1 type				
Modern	77 (55.0)	46.7–63.0	63 (45.0)	37.0–53.3
Ancestral	16 (55.2)	37.6–71.6	13 (44.8)	28.4–62.4

We also correlated each patient's clinical information (age, sex, TB history, contact history, and previous treatment outcome) and M. tuberculosis genotype with the PZA susceptibility testing result. Among the 169 patients with MDR-TB, 99 were male and the remaining 70 were female. The rate of PZA resistance was higher among the females (50.0%; 95% CI, 38.6 to 61.4%) than among the males (41.4%; 95% CI, 32.2 to 51.3%). Also, isolates from young patients (age, <20 years) showed higher rates of PZA^r^ (55.6%) than those from middle-aged or older patients (age >20 years) (39 to 45%). We did not find any significant correlation between a history of TB or a contact history and the PZA susceptibility patterns (Table 1). Patients who had previously failed a treatment regimen that included second-line drugs had a higher frequency (78.6%; 95% CI, 52.4 to 92.4%) of PZA^r^ than patients in the other categories. Genotyping of the M. tuberculosis isolates revealed that the frequency of PZA^r^ among Beijing lineage and modern lineage isolates was slightly higher than that among non-Beijing-type and ancestral-type isolates (Table 1).

### Correlation of PZA susceptibility and *pncA* gene mutation.

We sequenced the PZA resistance-associated gene (*pncA*) for all the isolates and got satisfactory sequences for 152. The results of the phenotypic PZA susceptibility test and the *pncA* sequencing results were concordant in 82.2% of cases, with 69 isolates with a sensitive *pncA* sequencing result being PZA^s^ by the phenotypic PZA assay and 56 isolates with a resistant *pncA* sequencing result being PZA^r^ by the phenotypic PZA assay (sensitivity, 90.3% [95% CI, 80.1 to 95.8%]; specificity, 76.7% [95% CI, 66.9 to 84.3%]). The sequencing result revealed that 68 isolates each harbored a single mutation, 9 had multiple mutations, 10 had a silent mutation (Ser65Ser), and 65 had no mutation (Table 2). When the sequencing result was considered the standard, it was found that 27 isolates showed a discrepancy between the sequencing result and the phenotypic PZA assay result. Six isolates without any mutation were PZA^r^ by the phenotypic assay, and 21 isolates were phenotypically PZA^s^, even though they had a mutation in the *pncA* gene. Three isolates carried multiple mutations but were phenotypically PZA^s^. Two isolates were phenotypically PZA^r^, even though they had the silent mutation (Ser65Ser).

**TABLE 2:** Correlation between phenotypic and genotypic PZA susceptibility results

Sequencing result	No. (%) of participants with the following MGIT PZA susceptibility assay result:		
	PZA^s^	PZA^r^	Total
No mutation	61 (93.8)	4 (6.2)	65
Single mutation	18 (26.5)	50 (73.5)	68
Silent mutation	8 (80.0)	2 (20.0)	10
Multiple mutation	3 (33.3)	6 (66.7)	9
Total	90	62	152

### *pncA* mutation profile and association with M. tuberculosis lineages.

A total of 87 (57.2%) of 152 isolates harbored a mutation in the *pncA* gene (Table 3). Nine isolates carried multiple mutations (double mutations in seven isolates, triple mutations in one isolate, and four mutations in one isolate), and the remaining 78 isolates harboring a mutation carried a single mutation (including a silent mutation) in the *pncA* gene. Mutations were found in both the putative promoter region (*n* = 7) and the *pncA* gene (*n* = 80). Different types of mutations, including insertion (ins) (*n* = 14), deletion (del) (*n* = 4), and point (*n* = 44) mutations, were found and were dispersed through the whole length of the *pncA* gene. When the silent and multiple mutations were included, 64 different types of mutations were found, and 12 of these were found in multiple isolates. The promoter mutations A(−11)G and A(−15)C were found in two and four cases, respectively. A deletion of a long segment (del of codon 37 [Cd37] to Cd187) was found in 3 isolates, a 2-base insertion (ins of GC at nucleotide 445) was found in 7 isolates, and a silent mutation (TCC-TCT, Ser65Ser) was found in 13 isolates (Table 3). We detected 27 new mutations that were not reported earlier in the literature.

**TABLE 3:** *pncA* mutation profile^*a*^

Nucleotide change	Amino acid change(s)	SNP location(s)	No. of cases	M. tuberculosis lineage(s)^*b*^	MGIT PZA susceptibility assay result^*b*^^,^^*c*^
Single mutation					
(−4) ins C^*d*^	NA	Promoter	1	EAI5	S
149 ins T^*d*^	Frameshift at Cd50	149	1	Beijing	S
156 ins T^*d*^	Frameshift at Cd53	156	1	Beijing	R
159 ins A^*d*^	Frameshift at Cd54	159	1	T1	S
166 ins G^*d*^	Frameshift at Cd56	166	1	Beijing	S
391 ins GG	Frameshift at Cd131	391	1	Beijing	R
392 ins GG	Frameshift at Cd131	392	1	EAI1-SOM	R
445 ins GC^*d*^	Frameshift at Cd149	445	6	LAM9 (6)	R (6)
450 ins GC^*d*^	Frameshift at Cd150	450	1	Beijing	R
518 ins G^*d*^	Frameshift at Cd173	518	1	Beijing	R
del 558 G^*d*^	Frameshift at Cd186	558	1	EAI	R
A(−11)G	NA	Promoter	2	Beijing (1), CAS1-Delhi (1)	R, R
A(−15)C	NA	Promoter	3	T1 (2), Orphan (1)	S, S, R
AAG to ATG	Lys96Met	287	1	LAM9	R
AAT to AGT^*d*^	Asn149Ser	446	1	T1	S
ACC to CCC	Thr47Pro	139	2	Beijing	R (2)
ACT to CCT	Thr76Pro	226	1	EAI1-SOM	R
AGC to AGG	Ser104Arg	312	2	Beijing (2)	R, S
ATT to ACT	Ile133Thr	398	1	Orphan	R
CAA to TAA	Gln122Stop	364	1	Beijing	S
CAC to CAG	His51Gln	153	1	T1	R
CAC to CCC	His51Pro	152	1	Beijing	R
CAG to AAG	Gln10Lys	28	1	LAM10-CAM	R
CAG to TAG	Gln10Stop	28	1	EAI1-SOM	R
CAT to CCT	His137Pro	410	1	T1	R
CAT to CGT	His71Arg	212	1	Beijing	R
CAT to CTT^*d*^	His137Leu	410	1	T1	R
CCG to TCG	Pro54Ser	160	1	Orphan	S
CCG to CTG	Pro54Leu	160	1	Orphan	R
CTG to CCG	Leu172Pro	515	1	T1	R
CGG to TGG^*d*^	Arg2Trp	4	1	LAM9	S
del 340 A^*d*^	Frameshift at Cd114	340	1	Beijing	R
del 112 to 561^*d*^	del of Cd37 to Cd187	112–561	3	Beijing (3)	R (3)
del 296 to 309^*d*^	Frameshift at Cd99	296–309	1	T1	R
GAC to AAC	Asp49Asn	145	1	Beijing	R
GAC to GCC	Asp12Ala	35	1	LAM9	R
GCC to ACC^*d*^	Ala28Thr	82	1	EAI3-IND	S
GCG to ACG	Ala171Thr	511	1	Beijing	R
GCG to GTG	Ala171Val	512	1	T1	S
GCG to GGG^*d*^	Ala171Gly	512	1	T1	S
GGC to GAC^*d*^	Gly105Asp	314	3	T1 (3)	R (2), S (1)
GGC to GAC	Gly24Asp	71	1	Beijing	R
GTG to GCG	Val139Ala	416	1	T1	R
GTG to ATG^*d*^	Val155Met	463	1	EAI-SOM	R
TAC to CAC	Tyr103His	307	1	T1	S
TAC to TAA	Tyr95Stop	285	1	T1	R
TAC to TAG	Tyr99Stop	297	3	Beijing	R (2), S (1)
TAC to TGC	Tyr103Arg	308	1	Orphan	R
TCC to TCT	Ser65Ser	195	10	CAS1-Delhi (10)	S (8), R (2)
TGT to GGT^*d*^	Cys138Gly	412	1	Orphan	R
TTC to GTC^*d*^	Phe58Val	172	1	Beijing	S
TTG to TGG	Leu4Trp	12	2	CAS1-Delhi (2)	R (2)
Multiple mutations					
TCC to TCT, 295 ins C,^*d*^ 430 ins C,^*d*^ 467 ins C^*d*^	Ser65Ser and frameshift at Cd99,144,156	195, 295, 430, 467	1	CAS1-Delhi	S
AAG to GAG, AGG to ACG, GAG to CAG^*d*^	Lys96Glu, Arg154Thr, Glu181Gln	286, 461, 541	1	EAI5	R
TCC to TCT, ACT to ATT	Ser65Ser, Thr76Ile	195, 227	1	CAS2	R
TGG to TGA, 445 ins GC	Trp119Stop, frameshift at Cd149	357, 445	1	LAM9	R
GAC to GCC, 178 ins C^*d*^	Asp12Ala, frameshift at Cd60	35, 178	1	LAM9	S
A(−15)C, TCG to CCG	NA, Ser67Pro	Promoter, 199	1	T1	R
GCG to GGG,^*d*^ GGC to GAC	Ala38Gly, Gly105Asp	113, 314	1	T1	R
TCC to TCT, GGT to AGT	Ser65Ser, Gly97Ser	195, 289	1	CAS1-Delhi	R
TAC to TGC, TGG to CGG	Tyr34Cys, Trp68Arg	101, 202	1	LAM9	S

a SNP, single nucleotide polymorphism; R, resistant; S, sensitive/susceptible; ins, insertion; del, deletion; NA, not applicable; nt, nucleotide; Cd, codon.

b Numbers in parentheses indicate the number of isolates of that type.

c Results of the MGIT assay for PZA susceptibility separated by a comma indicate the corresponding result for the M. tuberculosis lineage.

d New mutation.

We found an interesting correlation between the mutation in a specific M. tuberculosis genotype and the phenotypic outcome. It was found that some mutations in T1- and EAI-type isolates showed no phenotypic expression. For example, T1-type M. tuberculosis isolates with the promoter mutation A(−15)C were susceptible in the phenotypic PZA assay, whereas an Orphan-type isolate was PZA^r^. Some isolates sharing the same mutation were also grouped genetically. Deletion of a long DNA segment (del of Cd37 to Cd187) was found in only three Beijing-type isolates, and these isolates were phenotypically PZA^r^. The silent mutation Ser65Ser was found exclusively in 11 CAS1-Delhi-type isolates, and 2 of them were phenotypically PZA^r^. The insertion of GC at position 445 was found in seven LAM9-type isolates (including one isolate with a double mutation), and these isolates were also phenotypically PZA^r^ (Table 3).

### Treatment outcome and PZA susceptibility pattern.

Clinical information from up to 12 months of follow-up was available for 124 patients. We were unable to follow up 45 participants (2 refused to participate in follow-up, 12 died, and 31 were out of reach). The clinical outcome (smear and culture conversion timeline) was compared with the PZA susceptibility results for those participants for whom follow-up information was available. From this limited information, we did not find any significant correlation between PZA susceptibility and clinical outcome (Table 4). Smear and culture conversion occurred in, respectively, 121 (97.5%) and 108 (87%) cases of conversion. Except in three cases, all cases of smear and culture conversion occurred within 6 months of treatment. There was no significant difference in the conversion rate among participants infected with PZA^s^ and PZA^r^ isolates.

**TABLE 4:** Correlation of treatment outcome at 6 and 12 months of follow-up and PZA susceptibility^*a*^

Treatment outcome	PZA^s^		PZA^r^	
	No. (%) of participants	95% CI	No. (%) of participants	95% CI
Smear conversion				
Yes (*n* = 121)	67 (98.5)	92.1–99.7	54 (96.4)	87.9–99.0
No (*n* = 3)	1 (1.5)	0.3–7.9	2 (3.6)	1.0–12.1
Culture conversion				
Yes (*n* = 108)	60 (88.2)	78.5–93.9	48 (87.3)	76.0–93.7
No (*n* = 15)	8 (11.8)	6.1–21.5	7 (12.7)	6.3–24.0
Smear conversion at the following times (mo) after end of treatment:				
1–2	37 (55.3)	43.4–66.5	26 (48.1)	35.4–61.1
3–4	22 (32.8)	22.8–44.7	21 (38.9)	27.04–52.2
5–6	7 (10.4)	5.2–20.0	5 (9.3)	4.0–19.9
7–12	1 (1.5)	0.3–8.0	2 (3.7)	1.0–12.5
Culture conversion at the following times (mo) after end of treatment:				
1–2	33 (55)	42.5–66.9	23 (47.9)	34.5–61.7
3–4	23 (38.3)	27.1–51.0	20 (41.7)	28.8–55.7
5–6	3 (5.0)	1.7–13.7	3 (6.3)	2.1–16.8
7–12	1 (1.7)	0.3–8.8	2 (4.1)	1.1–14.0

a Data are for 124 participants.

## DISCUSSION

The aim of this analysis was to observe the PZA susceptibility pattern among MDR-TB patients in Bangladesh and compare the phenotypic PZA susceptibility with the *pncA* sequencing results. We also investigated the impact of PZA susceptibility on the treatment outcome for these patients.

In our analysis, we found that 45% of isolates (95% CI, 37.7 to 52.5%) were PZA^r^. This finding correlates with the findings of many other studies conducted in the United States (38.0%) (24), South Africa (39.3% and 52.1%) (26, 31), Belgium (43.4%) (13), Japan (53.0%) (32), Thailand (49%) (33), India (30.4%) (34), China (30.2%) (35), and Central Africa (50%) (36).

A history of TB and a TB contact history did not appear to have any significant impact on PZA susceptibility in our analysis, but isolates from patients who had previously failed treatment with a second-line drug regimen exhibited a higher frequency (78.6%; 95% CI, 52.4 to 92.4%) of PZA^r^ than isolates from patients who had been cured or who had completed treatment or those from patients who had defaulted (a patient whose treatment is interrupted for two consecutive months or more) or failed treatment with a first-line drug regimen (36 to 43%). Genotype analysis (spoligotyping) showed that none of the isolates was M. bovis, which is inherently PZA^r^. The frequency of PZA^r^ among modern- and/or Beijing-type M. tuberculosis isolates was slightly higher than that among ancient- and/or non-Beijing-type isolates. It was observed that about 43.0 to 57.0% of Beijing-, T-, LAM-, EAI-, and Orphan-type isolates were PZA^r^, but a comparatively low proportion of (30%) PZA^r^ isolates were of the CAS type (data not shown). Due to an insufficient sample size, we were unable to conclude that PZA susceptibility does not significantly associate with the genetic type of the M. tuberculosis isolates. However, a study from California also showed that PZA susceptibility does not correlate with the genetic lineage of M. tuberculosis (37).

The *pncA* gene of all isolates was sequenced, and a satisfactory sequence was found for 152. The concordance of the results of the phenotypic PZA assay and those of *pncA* sequencing was found to be 82.2% (Table 2). The discrepancy was due to 21 PZA^s^ isolates harboring a *pncA* mutation and 6 PZA^r^ isolates without any *pncA* mutation or with the silent mutation. We repeated the phenotypic PZA susceptibility test for about 10% (16/169) of the isolates, and the results remained similar to those of the initial test, which excludes the possibility of a technical error related to PZA susceptibility testing in MGITs. The finding that six PZA^r^ isolates did not have a *pncA* mutation (*n* = 4) or had the Ser65Ser silent mutation (*n* = 2) suggests there might be other mechanisms of phenotypic PZA^r^, such as insufficient drug uptake, an active efflux pump, or a mutation in the *panD* and *rpsA* genes (12, 17, 38). On the other hand, 21 phenotypically PZA^s^ isolates had a nonsynonymous *pncA* mutation. The outcome of repeat testing in MGITs for a subset of these isolates remained similar to that of the initial test. To investigate whether there was any lapse in *pncA* sequencing, we performed high-resolution melt (HRM) analysis of *pncA* for 12 of these isolates, and all of them were found to be PZA^r^. An explanation for such a finding could be the presence of heteroresistance or a mixed bacterial population (39). Another explanation could be the level of expression of the mutated gene. As the resistance phenotype results from a complex network of interactions of changes in various components, including genes, transcripts, and their products, some mutations may not result in the expression of a resistance phenotype (40). Therefore, we cannot rule out the possibility that these mutations cause a phenotype of low-level or no resistance. Overall, among the phenotypic PZA^r^ isolates, 90.3% (56/62) had a resistance-causing mutation in the *pncA* gene, which indicates that a *pncA* mutation is the major mechanism of PZA^r^ in M. tuberculosis. This finding is consistent with the findings of previous studies from Iran (71%) (41), Thailand (75%) (33), CDC (Atlanta, GA, USA) (84.6%) (42), China (91.4%) (43), India (97%) (44), and Belgium (98.3%) (13).

Overall, 64 different mutations (including a silent mutation) were found among 87 isolates (Table 3). The mutations were diverse and scattered throughout the gene, which included substitution (both promoter and coding region), insertion, and deletion of a single nucleotide or a large DNA segment, causing a frameshift. In this study, we found 27 new mutations which were not previously described in the literature (14, 35, 45–48). Each of 52 different mutations was found in one isolate, which indicates that M. tuberculosis isolates acquire diverse mutations to develop PZA resistance. Twelve mutations were found in multiple isolates (shared mutations). Ten isolates with shared mutations were also genetically clustered (i.e., they had the same spoligotype). It was found that 11 isolates having the Ser65Ser synonymous mutation were genetically of the CAS1-Delhi type. Studies have shown that the synonymous mutation Ser65Ser is predominantly found in some members of the CAS spoligotype family (34). A similar genetic grouping was found for three Beijing-type (del of Cd37 to Cd187), seven LAM9-type (ins of GC at nucleotide 445), three Beijing-type (Tyr99Stop), and two T1-type (Gly105Asp) isolates, and all of them were phenotypically resistant. On the other hand, two T1-type isolates sharing the promoter mutation A(−15)C were phenotypically PZA^s^. Our findings correlate with evidence of an association between a genotype cluster and the *pncA* mutation reported in different studies (13, 49–51). Also, the fact that isolates exhibit similar phenotypic properties strengthens evidence of transmission of these isolates.

The clinical outcome data for the different treatment regimens were available for 124 participants who were treated with either the WHO-recommended 24-month regimen or the Bangladesh regimen. Though the smear conversion rate was slightly higher among participants infected with PZA^s^ isolates than those infected with PZA^r^ isolates (98.5% versus 96.4%), the difference was not statistically significant (Table 4). A similar outcome was found in the case of the culture conversion rate. Except for three cases (two of which were caused by PZA^r^ isolates), all smear and culture conversions occurred within 6 months of treatment. These data reveal that the treatment outcome may not be heavily influenced by the PZA susceptibility status, which has also been described by earlier researchers (37).

The major limitation of our study is the fact that we were unable to obtain a satisfactory sequence of the *pncA* gene for about 10% of the isolates. Another limitation is the fact that we were not able to follow up almost one-fourth of our participants. This was because most of them were out of reach, 12 of them died, and 2 refused to participate in follow-up. As a result, we could not get a definitive picture of the clinical outcome with regard to PZA susceptibility.

Based on our findings and also evidence from other parts of the world, we can conclude that the rate of PZA resistance is fairly high in MDR-TB patients, and in most cases the resistance results from a mutation in the *pncA* gene. The results of phenotypic drug susceptibility testing (DST) have a very good concordance with the results of *pncA* sequencing. Considering the long turnaround time in the case of phenotypic DST, we can say that *pncA* sequencing is more feasible for the detection of PZA resistance. Furthermore, this information can be advantageous in developing future molecular diagnostic tests for drug-resistant TB. The prevalence of a high level of PZA resistance among MDR-TB patients in Bangladesh and many other countries, together with the fact that PZA susceptibility did not appear to have any significant effect on the final treatment outcome, provokes us to wonder about the effectiveness of the inclusion of the drug at its current dosage in the TB treatment regimen. It warrants further studies to analyze the MIC of PZA for isolates from MDR-TB patients and subsequent fine-tuning of the PZA dose in the MDR-TB treatment regimen.

## MATERIALS AND METHODS

### Study population.

Specimens were collected from the ongoing study Surveillance of MDR and Extensively Drug-Resistant Tuberculosis in Bangladesh, which was approved by the Research Review Committee (RRC) and the Ethical Review Committee (ERC) of the International Center for Diarrheal Disease Research, Bangladesh (ICDDR,B). In this surveillance, sputum specimens were collected from newly registered TB patients in 14 different hospitals across 12 districts covering all seven divisions of Bangladesh for MDR-TB surveillance. For surveillance for extensively drug-resistant TB (XDR-TB), specimens were collected from patients with known or suspected MDR-TB at three additional chest disease hospitals as well as the 14 hospitals mentioned earlier. Written informed consent, which was also approved by the RRC and the ERC, was obtained from the participants. Participants who did not give consent were not included in the study. The specimens were confirmed to be positive for the M. tuberculosis complex (MTBC) by an Xpert MTB/RIF assay. A total of 169 confirmed MDR M. tuberculosis isolates collected from 2011 to 2014 were included in this analysis. These did not include multiple isolates from any single participant.

### Specimen processing and culture.

All sputum specimens (*n* = 169) were digested and decontaminated by an *N*-acetyl-l-cysteine–sodium hydroxide (NALC-NaOH) decontamination method (39). Briefly, an equal volume of NALC-NaOH-Na citrate solution was added to the sputum specimen in a 50-ml centrifuge tube and vortexed for 10 to 20 s. The mixture was incubated at room temperature for 15 min, neutralized with phosphate-buffered saline (PBS; pH 6.80), mixed well by vortexing, and centrifuged at 3,000 × *g* (6-16k centrifuge; Sigma, UK) for 15 min. The supernatant was carefully decanted, and the pellet was resuspended in 1.0 ml PBS and, finally, inoculated in Lowenstein-Jensen (LJ) medium, which was incubated at 37°C for up to 8 weeks and checked weekly.

### DST.

The LJ proportion method for drug susceptibility testing (DST) was performed according to the standard protocol described previously (39). Drug-containing LJ media were prepared so that they had final concentrations of INH of 0.2 μg/ml, RIF of 40.0 μg/ml, STR of 4.0 μg/ml, and EMB of 2.0 μg/ml. Stock solutions of drugs were prepared from reference powders, and all media were prepared in-house and tested for sterility and performance. Briefly, a 1.0 McFarland standard mycobacterial suspension was prepared in sterile distilled water from the freshly grown colony and serially diluted to four different concentrations of cells (10^−1^ to 10^−4^ cells/ml). The suspension (10.0 μl) was inoculated onto LJ slants with and without drugs with a platinum loop with an internal diameter of 3.0 mm calibrated to 10 μl. LJ slants were incubated at 37°C, and depending on the growth on control media, the results were read at day 28, at day 35, and, finally, at day 42. The criterion of resistance was colony growth on the drug-containing media at a level equal to or 1% above that on drug-free media. A susceptible strain, H37Rv (ATCC), and our SB256 strain, determined to be resistant to STR, INH, RIF, and EMB in the laboratory (which was confirmed by the LJ test method, DST in a mycobacterium growth indicator tube [MGIT], and sequencing of the gene responsible for resistance), were used for quality control.

### Phenotypic PZA susceptibility assay.

The phenotypic PZA susceptibility assay was done by using a Bactec MGIT 960 PZA kit (BD, Franklin Lakes, NJ, USA) following the manufacturer's instructions. Briefly, pure colonies from the LJ slant were scraped off and suspended in normal saline, and the suspension was adjusted to a 0.5 McFarland standard by visual comparison. The adjusted suspension was diluted to a 1:5 ratio and used as the inoculum for antimicrobial susceptibility testing (AST). The inoculum used for AST was diluted 10 times and used as a growth control (GC). Two 7.0-ml MGITs were prepared for each test isolate, and one was labeled GC and the other one was labeled PZA. In each tube, 0.8 ml of Bactec MGIT 960 PZA supplement was added aseptically, and, additionally, 100 μl of PZA drug solution (final concentration, 100 μg/ml) was added to the PZA tube. Finally, 0.5 ml of the GC and AST inocula were inoculated into the respective tubes and the tubes were loaded into the Bactec MGIT 960 instrument. The instrument continuously monitored the susceptibility test until a susceptible or resistant determination was made. For phenotypic PZA susceptibility testing, a susceptible strain, H37Rv (ATCC), was included as the sensitive control.

### DNA extraction.

DNA was extracted from a 3- to 4-week-old culture grown on an LJ slant. Pure colonies were scraped off and dissolved in sterile distilled water to prepare a suspension with a turbidity equal to that of a 0.5 McFarland standard. Two hundred microliters of the suspension was transferred to a 2.0-ml screw-cap tube and heat inactivated at 95°C for 30 min. DNA was isolated from the heat-inactivated suspension using a QIAamp DNA minikit (Qiagen Inc., Valencia, CA, USA) following the manufacturer's instruction.

### TbD1 analysis.

PCR based M. tuberculosis-specific deletion (TbD1) analysis was performed using an internal primer set (forward primer int-F [5′-CGTTCAACCCCAAACAGGTA-3′] and reverse primer int-R [5′-AATCGAACTCGTGGAACACC-3′]) and a flanking primer set (forward primer flnk-F [5′-CTACCTCATCTTCCGGCCA-3′] and reverse primer flnk-R [5′-CATAGATCCCGGACATGGTG-3′]). Amplification of the internal and flanking regions was performed separately. Each reaction mixture was prepared in a total volume of 15.0 μl and contained 3.0 μl of 5× Green PCR buffer (Promega, USA), 1.5 mM MgCl_2_, 0.2 mM deoxynucleoside triphosphate (dNTP) mix, 1.2 μM each forward and reverse primer, 0.375 U of *Taq* polymerase, and 2.0 μl of template DNA (approximately 20.0 ng/μl). PCR amplification was performed on a Veriti 96-well thermal cycler (Applied Biosystems) with initial denaturation at 95°C for 1 min, followed by 35 cycles of denaturation at 95°C for 30 s, annealing at 56°C for 1 min 30 s, and extension at 72°C for 4 min and a final extension at 72°C for 5 min. The amplified PCR products were analyzed by agarose gel electrophoresis with 1.5% analytical-grade agarose (Promega, USA).

### Spoligotyping.

Spoligotyping was performed according to the standard protocol (52). The presence or absence of 43 variable spacers in the direct repeat region of M. tuberculosis was determined by using primers DRa (5′-GGTTTTGGGTCTGACGAC-3′, which was 5′ biotinylated) and DRb (5′-CCGAGAGGGGACGGAAAC-3′). Each reaction mixture was prepared in a total volume of 20.0 μl and contained 2.0 μl of 10× Super *Tth* buffer, 0.2 mM dNTP mix, 1.6 μM each forward and reverse primers, 0.2 U of *Taq* polymerase, and 2.0 μl of template DNA. PCR amplification was performed on a Veriti 96-well thermal cycler (Applied Biosystems) with an initial denaturation at 96°C for 3 min, followed by 35 cycles of denaturation at 96°C for 1 min, annealing at 55°C for 1 min, and extension at 72°C for 1 min and a final extension of 72°C for 5 min.

The resulting amplicons were hybridized with a commercially available membrane (Isogen Bioscience BV, Bilthoven, Netherlands). The membrane contains 43 covalently linked synthetic oligonucleotides corresponding to 43 spacers arranged in parallel rows. The hybridization patterns were detected by enhanced chemiluminescence (ECL; Amersham, UK). The detected bands were converted to 43 binary codes. Spoligotype data were analyzed by SITVITWEB, an online tool of the Institut Pasteur de Guadeloupe (http://www.pasteur-guadeloupe.fr:8081/SITVIT_ONLINE/).

### Sanger sequencing of *pncA* gene.

We sequenced the *pncA* gene of all isolates. The sequencing was done according to procedures described earlier (53). Briefly, the *pncA* gene was amplified by PCR using forward primer 5′-GGTCATGTTCGCGATCGTCG-3′ and reverse primer 5′-ACAGTTCATCCCGGTTCGGC-3′. Each 25-μl PCR mixture contained 12.5 μl HotStar *Taq* master mix (Qiagen Inc., Valencia, CA, USA), 0.15 μl each of 50 μM forward and reverse primers, 7.2 μl of nuclease-free water, and 5 μl of genomic DNA. PCR was performed on a MyCycler instrument (Bio-Rad, Hercules, CA, USA) with an initial denaturation at 95°C for 15 min, followed by 35 cycles of denaturation at 95°C for 30 s, annealing at 60°C for 30 s, and extension at 72°C for 30 s and a final extension at 72°C for 7 min. PCR products were analyzed on 2% agarose gels and were purified using a MinElute PCR purification kit (Qiagen Inc., Valencia, CA, USA). The purified PCR products were sequenced at ICDDR,B using an ABI 3500xL genetic analyzer (Applied Biosystems). The resulting raw sequences were analyzed by Chromas software (version 2.33) and aligned with the WT sequence using ClustalW multiple-sequence alignment incorporated into BioEdit sequence alignment editor software (version 7.2.6).

### Participant follow-up.

All the participants were followed up after 6 and 12 months from the beginning of their enrollment in the study. During the follow-up, clinical outcome data in terms of smear microscopy and culture conversion results and the treatment regimen, as well as the documented treatment outcome at the time of follow-up, were collected from the participants and also from the hospital records.

### Data storage and analysis.

Information and laboratory data for all participants were entered in Microsoft Excel 2013 software and were imported into SPSS (version 20; IBM) software. Frequency distributions were analyzed by use of the SPSS (version 20) software. Spoligotyping patterns were converted into 43 binary codes, recorded in Excel software, and uploaded to the SITVITWEB database. The 95% confidence intervals were calculated by use of the OPENEPI website (http://www.openepi.com/Proportion/Proportion.htm).
